# *trans*-Tetra­carbonyl­bis­[tris­(3-fluoro­phen­yl)phosphane]chromium(0)

**DOI:** 10.1107/S1600536811045284

**Published:** 2011-11-02

**Authors:** M. N. Norlidah, M. Y. Yazid, Omar bin Shawkataly, Mohd Mustaqim Rosli, Hoong-Kun Fun

**Affiliations:** a Faculty of Industrial Science and Technology, Universiti Malaysia Pahang, Gambang 26300, Pahang, Malaysia; b Chemical Sciences Programme, School of Distance Education, Universiti Sains Malaysia, 11800 USM, Penang, Malaysia; c X-ray Crystallography Unit, School of Physics, Universiti Sains Malaysia, 11800 USM, Penang, Malaysia

## Abstract

In the title compound, [Cr(C_18_H_12_F_3_P)_2_(CO)_4_], the Cr atom is octa­hedrally coordinated by four carbonyl ligands and the two tertiary phosphanes, which are *trans* to each other. The three benzene rings in one phosphane ligand make dihedral angles of 53.50 (9), 75.51 (10) and 80.63 (10)° with each other, while in the other ligand these angles are 51.92 (10), 78.56 (11) and 86.80 (10)°. C—H⋯O and C—H⋯F inter­actions link the mol­ecules into a three-dimensional network. Each of the F atoms is disordered over two positions with refined occupancies of 0.944 (3):0.056 (3), 0.702 (4):0.298 (4), 0.829 (4):0.171 (4), 0.567 (4):0.433 (4), 0.545 (4):0.455 (4) and 0.920 (4):0.080 (4).

## Related literature

For related structures, see: Bennett *et al.* (2004 ▶); Brunet *et al.* (2002 ▶); Preston *et al.* (1972 ▶); Shawkataly *et al.* (1996 ▶, 2009 ▶); Norlidah *et al.* (2011 ▶). A search of the Cambridge Structural Database (Allen, 2002 ▶) revealed 113 complexes of carbonyl­chromium complexes with bis-phosphanes. For hydrogen-bond motifs, see: Bernstein *et al.* (1995 ▶). For the stability of the temperature controller used in the data collection, see: Cosier & Glazer (1986 ▶).
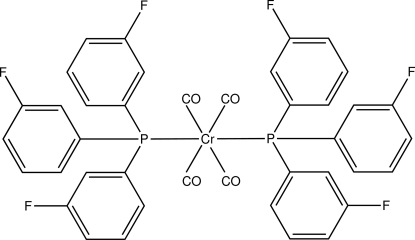


## Experimental

### 

#### Crystal data

[Cr(C_18_H_12_F_3_P)_2_(CO)_4_]
*M* *_r_* = 796.53
Monoclinic, 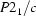

*a* = 12.1675 (2) Å
*b* = 18.5362 (3) Å
*c* = 15.4084 (2) Å
β = 90.448 (1)°
*V* = 3475.09 (9) Å^3^
*Z* = 4
Mo *K*α radiation
μ = 0.50 mm^−1^
*T* = 100 K
0.26 × 0.25 × 0.19 mm

#### Data collection

Bruker SMART APEXII CCD area-detector diffractometer
Absorption correction: multi-scan (*SADABS*; Bruker, 2009 ▶) *T*_min_ = 0.881, *T*_max_ = 0.912
104037 measured reflections
14097 independent reflections
9496 reflections with *I* > 2σ(*I*)
*R*_int_ = 0.054

#### Refinement

*R*[*F* ^2^ > 2σ(*F* ^2^)] = 0.056
*wR*(*F* ^2^) = 0.139
*S* = 1.21
14097 reflections
528 parameters
4 restraints
H-atom parameters constrained
Δρ_max_ = 1.19 e Å^−3^
Δρ_min_ = −1.41 e Å^−3^

### 

Data collection: *APEX2* (Bruker, 2009 ▶); cell refinement: *SAINT* (Bruker, 2009 ▶); data reduction: *SAINT*; program(s) used to solve structure: *SHELXTL* (Sheldrick, 2008 ▶); program(s) used to refine structure: *SHELXTL*; molecular graphics: *SHELXTL*; software used to prepare material for publication: *SHELXTL* and *PLATON* (Spek, 2009 ▶).

## Supplementary Material

Crystal structure: contains datablock(s) I, global. DOI: 10.1107/S1600536811045284/is2790sup1.cif

Structure factors: contains datablock(s) I. DOI: 10.1107/S1600536811045284/is2790Isup2.hkl

Additional supplementary materials:  crystallographic information; 3D view; checkCIF report

## Figures and Tables

**Table 1:** Selected bond lengths (Å)

Cr1—C37	1.8890 (18)
Cr1—C38	1.882 (2)
Cr1—C39	1.888 (2)
Cr1—C40	1.8885 (19)
Cr1—P1	2.3333 (5)
Cr1—P2	2.3320 (5)

**Table 2:** Hydrogen-bond geometry (Å, °)

*D*—H⋯*A*	*D*—H	H⋯*A*	*D*⋯*A*	*D*—H⋯*A*
C2—H2*A*⋯O2^i^	0.95	2.52	3.105 (2)	120
C4—H4*A*⋯F1^ii^	0.95	2.41	3.292 (2)	153
C10—H10*A*⋯F4^iii^	0.95	2.53	3.271 (3)	135
C28—H28*A*⋯F5^iv^	0.95	2.33	3.069 (3)	135
C34—H34*A*⋯F3^v^	0.95	2.32	3.191 (3)	153

Symmetry codes: (i) 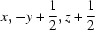; (ii) 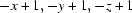; (iii) 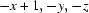; (iv) 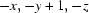; (v) 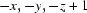.

**Fig. 1.**
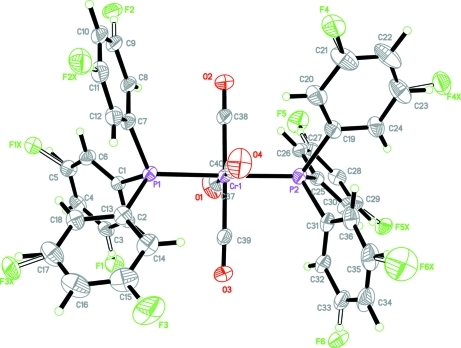
The molecular structure of the title compound, showing 50% probability displacement ellipsoids. Hydrogen atoms are shown as spheres of arbitrary radius.

**Fig. 2.**
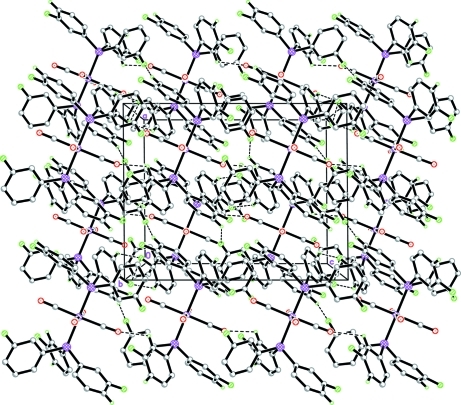
The packing diagram of the title compound. Dashed lines indicate hydrogen bonds. H atoms not involved in the hydrogen bond interactions have been omitted for clarity.

## supplementary crystallographic information

### Comment

The bonding characteristics of metal carbonyls with a phosphine ligand in phosphine-substituted metal carbonyls are of interest. Several crystal structures of phosphine-substituted group 6 carbonyls with *trans* coordination of phosphine have been reported (Brunet *et al.*, 2002; Bennett *et al.*, 2004).

In the title compound where the fluorine atom is in the *meta* position on each benzene ring (Fig. 1), the Cr—P bond lengths, with an average value of 2.3327 Å (Table 1), are relatively short despite the presence of the bulky phosphine ligand, compared to the average value of 2.3656 (16) Å in the complex *trans*-Cr(CO)_4_(PPh_3_)_2_ (Bennett *et al.*, 2004). We have previously reported the C_40_H_24_CrF_6_O_4_P_2_ but the fluorine atom is in the *para* position whereby the average of Cr—P bond lengths is almost similar (Norlidah *et al.*, 2011).

All six fluorine atoms in the title compound are disordered over two positions with the final refined occupancies being 0.944 (3):0.056 (3) (F1:F1X), 0.702 (4):0.298 (4) (F2:F2X), 0.829 (4):0.171 (4) (F3:F3X), 0.567 (4):0.433 (4) (F4:F4X), 0.545 (4):0.455 (4) (F5:F5X) and 0.920 (4):0.080 (4) (F6:F6X) (Fig. 1). The dihedral angles of the three benzene rings attached to the P1 atom are 53.50 (9)° (between C1—C6 & C7—C12), 80.63 (10)° (between C1—C6 & C13—C18) and 75.51 (10)° (between C7—C12 & C13—C18) and the dihedral angles of the three benzene ring attached to the P2 atom are 86.80 (10)° (between C19—C24 & C25—C30), 78.56 (11)° (between C19—C24 & C31—C36) and 51.92 (10)° (between C25—C30 & C31—C36).

C4—H4A···F1^ii^ interaction showing a *R*^2^_2_(8) hydrogen ring motif and other intermolecular interactions C2—H2A···O2^i^, C10—C10A···F4^iii^, C28—H28A···F5^iv^ and C34—H34A···F3^v^ (Table 2) linked the molecules to form an infinite three-dimensional network (Fig. 2).

### Experimental

All manipulations were performed under a dry, oxygen-free nitrogen atmosphere using standard Schlenk techniques. All solvents were dried over sodium under dry oxygen-free nitrogen. Chromium hexacarbonyl (200 mg, 0.909 mmol) and tris-(3-flurophenyl)-phosphine (301.8 mg, 0.9542 mmol) in 30 ml of pet ether (100–130 °C) was refluxed for 12 h. Suitable single crystals were obtained by solvent-solvent diffusion in a mixture of dichloromethane/methanol.

### Refinement

All six fluorine atoms are disordered over two position with the final refined occupancies being 0.944 (3):0.056 (3) (F1:F1X), 0.702 (4):0.298 (4) (F2:F2X), 0.829 (4):0.171 (4) (F3:F3X), 0.567 (4):0.433 (4) (F4:F4X), 0.545 (4):0.455 (4) (F5:F5X) and 0.920 (4):0.080 (4) (F6:F6X). C5—F1X, C11—F2X, C23—F4X and C35—F6X distances were restrained to be around 1.399 (1) Å. F1X and F6X were refined isotropically due to the very small occupancies. All hydrogen atoms were positioned geometrically and refined using a riding model with C—H = 0.95 Å and *U*_iso_(H) = 1.2*U*_eq_(C).

### Crystal data

**Table:**

[Cr(C_18_H_12_F_3_P)_2_(CO)_4_]	*F*(000) = 1616
*M**_r_* = 796.53	*D*_x_ = 1.522 Mg m^−^^3^
Monoclinic, *P*2_1_/*c*	Mo *K*α radiation, λ = 0.71073 Å
Hall symbol: -P 2ybc	Cell parameters from 9990 reflections
*a* = 12.1675 (2) Å	θ = 2.4–33.4°
*b* = 18.5362 (3) Å	µ = 0.50 mm^−^^1^
*c* = 15.4084 (2) Å	*T* = 100 K
β = 90.448 (1)°	Block, yellow
*V* = 3475.09 (9) Å^3^	0.26 × 0.25 × 0.19 mm
*Z* = 4	

### Data collection

**Table:**

Bruker SMART APEXII CCD area-detector diffractometer	14097 independent reflections
Radiation source: fine-focus sealed tube	9496 reflections with *I* > 2σ(*I*)
graphite	*R*_int_ = 0.054
φ and ω scans	θ_max_ = 34.0°, θ_min_ = 1.7°
Absorption correction: multi-scan (*SADABS*; Bruker, 2009)	*h* = −19→18
*T*_min_ = 0.881, *T*_max_ = 0.912	*k* = −29→29
104037 measured reflections	*l* = −24→22

### Refinement

**Table:**

Refinement on *F*^2^	Primary atom site location: structure-invariant direct methods
Least-squares matrix: full	Secondary atom site location: difference Fourier map
*R*[*F*^2^ > 2σ(*F*^2^)] = 0.056	Hydrogen site location: inferred from neighbouring sites
*wR*(*F*^2^) = 0.139	H-atom parameters constrained
*S* = 1.21	*w* = 1/[σ^2^(*F*_o_^2^) + (0.041*P*)^2^ + 2.0091*P*] where *P* = (*F*_o_^2^ + 2*F*_c_^2^)/3
14097 reflections	(Δ/σ)_max_ < 0.001
528 parameters	Δρ_max_ = 1.19 e Å^−^^3^
4 restraints	Δρ_min_ = −1.41 e Å^−^^3^

### Special details

**Table:**

Experimental. The crystal was placed in the cold stream of an Oxford Cryosystems Cobra open-flow nitrogen cryostat (Cosier & Glazer, 1986) operating at 100.0 (1) K.
Geometry. All e.s.d.'s (except the e.s.d. in the dihedral angle between two l.s. planes) are estimated using the full covariance matrix. The cell e.s.d.'s are taken into account individually in the estimation of e.s.d.'s in distances, angles and torsion angles; correlations between e.s.d.'s in cell parameters are only used when they are defined by crystal symmetry. An approximate (isotropic) treatment of cell e.s.d.'s is used for estimating e.s.d.'s involving l.s. planes.
Refinement. Refinement of *F*^2^ against ALL reflections. The weighted *R*-factor *wR* and goodness of fit *S* are based on *F*^2^, conventional *R*-factors *R* are based on *F*, with *F* set to zero for negative *F*^2^. The threshold expression of *F*^2^ > σ(*F*^2^) is used only for calculating *R*-factors(gt) *etc*. and is not relevant to the choice of reflections for refinement. *R*-factors based on *F*^2^ are statistically about twice as large as those based on *F*, and *R*- factors based on ALL data will be even larger.

### Fractional atomic coordinates and isotropic or equivalent isotropic displacement parameters (Å^2^)

**Table:**

	*x*	*y*	*z*	*U*_iso_*/*U*_eq_	Occ. (<1)
Cr1	0.24363 (2)	0.212464 (14)	0.238820 (18)	0.01984 (6)	
P1	0.42089 (4)	0.20679 (2)	0.29728 (3)	0.02068 (9)	
P2	0.06713 (4)	0.20669 (2)	0.17916 (3)	0.02217 (9)	
F1	0.40001 (11)	0.43367 (7)	0.47940 (9)	0.0403 (4)	0.944 (3)
F1X	0.7317 (4)	0.3942 (11)	0.3182 (13)	0.032 (6)*	0.056 (3)
F2	0.66968 (14)	0.20076 (10)	0.02992 (11)	0.0356 (5)	0.702 (4)
F2X	0.6755 (4)	−0.00403 (16)	0.1916 (3)	0.0491 (15)	0.298 (4)
F3	0.30991 (17)	0.03694 (11)	0.54658 (15)	0.0689 (8)	0.829 (4)
F3X	0.6380 (7)	0.1297 (5)	0.5745 (5)	0.049 (3)	0.171 (4)
F4	0.1674 (2)	0.03788 (14)	−0.07513 (17)	0.0515 (9)	0.567 (4)
F4X	−0.1622 (2)	0.13733 (19)	−0.0959 (2)	0.0514 (11)	0.433 (4)
F5	0.0764 (2)	0.44328 (14)	0.02129 (18)	0.0500 (8)	0.545 (4)
F5X	−0.2518 (2)	0.39483 (15)	0.16862 (18)	0.0355 (8)	0.455 (4)
F6	−0.17803 (11)	0.19241 (8)	0.44892 (9)	0.0413 (5)	0.920 (4)
F6X	−0.175 (2)	−0.0154 (6)	0.2925 (19)	0.090 (10)*	0.080 (4)
O1	0.24251 (14)	0.37600 (8)	0.24574 (11)	0.0428 (4)	
O2	0.33543 (13)	0.23794 (10)	0.05910 (10)	0.0415 (4)	
O3	0.14885 (12)	0.22402 (10)	0.41964 (10)	0.0409 (4)	
O4	0.24657 (14)	0.04846 (8)	0.23311 (13)	0.0494 (4)	
C1	0.48190 (14)	0.29334 (9)	0.33036 (11)	0.0208 (3)	
C2	0.41993 (15)	0.33288 (9)	0.38965 (11)	0.0234 (3)	
H2A	0.3509	0.3154	0.4090	0.028*	
C3	0.46066 (16)	0.39758 (10)	0.41947 (12)	0.0278 (4)	
H3A	0.4192	0.4233	0.4613	0.033*	0.056 (3)
C4	0.55815 (17)	0.42703 (10)	0.39197 (13)	0.0310 (4)	
H4A	0.5826	0.4727	0.4124	0.037*	
C5	0.61897 (17)	0.38759 (11)	0.33359 (13)	0.0320 (4)	
H5A	0.6870	0.4063	0.3136	0.038*	0.944 (3)
C6	0.58257 (16)	0.32087 (11)	0.30334 (13)	0.0289 (4)	
H6A	0.6265	0.2940	0.2642	0.035*	
C7	0.52277 (14)	0.16517 (10)	0.22598 (12)	0.0252 (3)	
C8	0.55869 (15)	0.20189 (11)	0.15205 (13)	0.0282 (4)	
H8A	0.5341	0.2495	0.1401	0.034*	
C9	0.63082 (16)	0.16734 (13)	0.09669 (14)	0.0350 (5)	
H9A	0.6565	0.1929	0.0474	0.042*	0.298 (4)
C10	0.66706 (16)	0.09799 (13)	0.10953 (15)	0.0382 (5)	
H10A	0.7160	0.0752	0.0704	0.046*	
C11	0.62923 (18)	0.06403 (12)	0.18087 (15)	0.0403 (5)	
H11A	0.6528	0.0159	0.1909	0.048*	0.702 (4)
C12	0.55879 (16)	0.09447 (11)	0.24012 (14)	0.0307 (4)	
H12A	0.5353	0.0680	0.2894	0.037*	
C13	0.44428 (16)	0.15630 (9)	0.39854 (13)	0.0264 (3)	
C14	0.36388 (19)	0.11232 (11)	0.43427 (15)	0.0359 (4)	
H14A	0.2953	0.1054	0.4055	0.043*	
C15	0.3861 (2)	0.07881 (13)	0.51269 (18)	0.0488 (6)	
H15A	0.3306	0.0493	0.5373	0.059*	0.171 (4)
C16	0.4836 (3)	0.08589 (13)	0.55666 (17)	0.0506 (6)	
H16A	0.4959	0.0622	0.6106	0.061*	
C17	0.5632 (2)	0.12834 (12)	0.52011 (16)	0.0440 (6)	
H17A	0.6321	0.1335	0.5488	0.053*	0.829 (4)
C18	0.54504 (17)	0.16367 (11)	0.44241 (14)	0.0322 (4)	
H18A	0.6011	0.1931	0.4186	0.039*	
C19	0.04474 (16)	0.15870 (10)	0.07525 (13)	0.0286 (4)	
C20	0.12532 (19)	0.11578 (11)	0.03810 (15)	0.0370 (5)	
H20A	0.1944	0.1095	0.0662	0.044*	
C21	0.1035 (3)	0.08208 (13)	−0.04079 (17)	0.0517 (7)	
H21A	0.1588	0.0526	−0.0657	0.062*	0.433 (4)
C22	0.0051 (3)	0.08976 (12)	−0.08393 (17)	0.0535 (7)	
H22A	−0.0094	0.0672	−0.1382	0.064*	
C23	−0.0688 (2)	0.13127 (13)	−0.04406 (16)	0.0501 (6)	
H23A	−0.1379	0.1366	−0.0725	0.060*	0.567 (4)
C24	−0.05633 (18)	0.16659 (11)	0.03242 (14)	0.0349 (4)	
H24A	−0.1135	0.1953	0.0558	0.042*	
C25	0.00110 (14)	0.29297 (9)	0.15190 (11)	0.0224 (3)	
C26	0.06208 (15)	0.34021 (10)	0.10104 (12)	0.0269 (4)	
H26A	0.1337	0.3271	0.0826	0.032*	
C27	0.01821 (18)	0.40595 (12)	0.07759 (13)	0.0333 (4)	
H27A	0.0604	0.4373	0.0422	0.040*	0.455 (4)
C28	−0.08477 (18)	0.42789 (11)	0.10357 (13)	0.0327 (4)	
H28A	−0.1129	0.4740	0.0884	0.039*	
C29	−0.14483 (17)	0.38031 (12)	0.15219 (14)	0.0336 (4)	
H29A	−0.2165	0.3939	0.1699	0.040*	0.545 (4)
C30	−0.10467 (16)	0.31307 (11)	0.17641 (14)	0.0307 (4)	
H30A	−0.1488	0.2811	0.2094	0.037*	
C31	−0.03148 (15)	0.16095 (10)	0.24952 (13)	0.0270 (4)	
C32	−0.07029 (15)	0.19584 (11)	0.32351 (13)	0.0292 (4)	
H32A	−0.0506	0.2445	0.3355	0.035*	
C33	−0.13789 (16)	0.15805 (12)	0.37881 (14)	0.0347 (4)	
H33A	−0.1658	0.1823	0.4283	0.042*	0.080 (4)
C34	−0.16684 (18)	0.08738 (13)	0.36618 (17)	0.0428 (6)	
H34A	−0.2132	0.0630	0.4059	0.051*	
C35	−0.12655 (19)	0.05288 (12)	0.29403 (17)	0.0433 (6)	
H35A	−0.1445	0.0037	0.2842	0.052*	0.920 (4)
C36	−0.06001 (17)	0.08897 (11)	0.23521 (15)	0.0344 (4)	
H36A	−0.0340	0.0646	0.1852	0.041*	
C37	0.24234 (15)	0.31430 (10)	0.24311 (12)	0.0264 (3)	
C38	0.30195 (15)	0.22475 (11)	0.12700 (13)	0.0278 (4)	
C39	0.18387 (15)	0.21704 (10)	0.35137 (13)	0.0270 (4)	
C40	0.24578 (16)	0.11064 (10)	0.23508 (14)	0.0309 (4)	

### Atomic displacement parameters (Å^2^)

**Table:**

	*U*^11^	*U*^22^	*U*^33^	*U*^12^	*U*^13^	*U*^23^
Cr1	0.01851 (12)	0.01680 (11)	0.02425 (13)	−0.00013 (10)	0.00279 (9)	−0.00305 (10)
P1	0.01922 (19)	0.01744 (18)	0.0254 (2)	0.00003 (15)	0.00260 (15)	−0.00167 (15)
P2	0.0202 (2)	0.01820 (19)	0.0281 (2)	−0.00114 (15)	0.00099 (16)	−0.00416 (16)
F1	0.0426 (8)	0.0379 (7)	0.0403 (8)	0.0025 (6)	0.0016 (6)	−0.0207 (6)
F2	0.0316 (9)	0.0462 (11)	0.0292 (9)	0.0083 (7)	0.0095 (7)	−0.0004 (7)
F2X	0.047 (3)	0.046 (3)	0.055 (3)	0.008 (2)	−0.010 (2)	−0.020 (2)
F3	0.0667 (14)	0.0577 (13)	0.0826 (15)	−0.0146 (10)	0.0198 (11)	0.0404 (11)
F3X	0.040 (5)	0.071 (6)	0.035 (4)	0.005 (4)	−0.012 (3)	0.019 (4)
F4	0.0471 (15)	0.0499 (15)	0.0573 (16)	0.0245 (12)	−0.0056 (12)	−0.0319 (12)
F4X	0.0410 (19)	0.059 (2)	0.054 (2)	−0.0019 (15)	−0.0088 (15)	−0.0210 (17)
F5	0.0517 (16)	0.0406 (14)	0.0579 (17)	−0.0084 (12)	0.0051 (12)	0.0287 (12)
F5X	0.0243 (13)	0.0437 (16)	0.0387 (15)	0.0119 (11)	0.0054 (10)	−0.0001 (12)
F6	0.0325 (8)	0.0524 (9)	0.0393 (8)	−0.0061 (6)	0.0121 (6)	0.0051 (6)
O1	0.0566 (10)	0.0213 (6)	0.0502 (10)	0.0026 (6)	−0.0201 (8)	−0.0031 (6)
O2	0.0335 (8)	0.0637 (11)	0.0272 (7)	0.0015 (7)	0.0065 (6)	−0.0040 (7)
O3	0.0296 (7)	0.0637 (11)	0.0294 (7)	−0.0003 (7)	0.0068 (6)	0.0015 (7)
O4	0.0523 (10)	0.0201 (7)	0.0757 (13)	0.0019 (7)	−0.0073 (9)	−0.0043 (7)
C1	0.0218 (7)	0.0186 (7)	0.0221 (7)	−0.0010 (6)	0.0009 (6)	−0.0011 (6)
C2	0.0255 (8)	0.0235 (8)	0.0213 (8)	0.0004 (6)	0.0007 (6)	−0.0014 (6)
C3	0.0342 (10)	0.0252 (8)	0.0239 (8)	0.0036 (7)	−0.0032 (7)	−0.0063 (7)
C4	0.0414 (11)	0.0229 (8)	0.0287 (9)	−0.0059 (7)	−0.0079 (8)	−0.0016 (7)
C5	0.0329 (10)	0.0324 (10)	0.0308 (10)	−0.0136 (8)	0.0014 (8)	−0.0001 (8)
C6	0.0258 (9)	0.0307 (9)	0.0302 (9)	−0.0077 (7)	0.0054 (7)	−0.0056 (7)
C7	0.0194 (7)	0.0254 (8)	0.0310 (9)	0.0016 (6)	−0.0004 (6)	−0.0086 (7)
C8	0.0211 (8)	0.0317 (9)	0.0319 (9)	0.0012 (7)	0.0033 (7)	−0.0092 (7)
C9	0.0207 (8)	0.0492 (12)	0.0351 (10)	0.0013 (8)	0.0040 (7)	−0.0161 (9)
C10	0.0230 (9)	0.0509 (13)	0.0407 (12)	0.0088 (8)	−0.0006 (8)	−0.0223 (10)
C11	0.0303 (10)	0.0433 (12)	0.0471 (13)	0.0124 (9)	−0.0091 (9)	−0.0221 (10)
C12	0.0274 (9)	0.0286 (9)	0.0361 (10)	0.0055 (7)	−0.0038 (7)	−0.0095 (8)
C13	0.0295 (9)	0.0178 (7)	0.0318 (9)	0.0030 (6)	0.0011 (7)	0.0011 (6)
C14	0.0387 (11)	0.0231 (9)	0.0459 (12)	−0.0024 (8)	0.0040 (9)	0.0079 (8)
C15	0.0642 (17)	0.0296 (11)	0.0528 (15)	0.0000 (11)	0.0095 (12)	0.0161 (10)
C16	0.0795 (19)	0.0319 (11)	0.0402 (13)	0.0108 (12)	−0.0036 (12)	0.0115 (9)
C17	0.0589 (15)	0.0332 (11)	0.0398 (12)	0.0103 (10)	−0.0130 (11)	0.0032 (9)
C18	0.0344 (10)	0.0271 (9)	0.0349 (10)	0.0040 (8)	−0.0035 (8)	0.0009 (8)
C19	0.0322 (9)	0.0215 (8)	0.0321 (9)	−0.0032 (7)	−0.0012 (7)	−0.0066 (7)
C20	0.0462 (12)	0.0257 (9)	0.0390 (11)	0.0056 (8)	−0.0037 (9)	−0.0124 (8)
C21	0.0818 (19)	0.0291 (11)	0.0442 (13)	0.0111 (12)	−0.0065 (13)	−0.0176 (10)
C22	0.092 (2)	0.0282 (11)	0.0399 (13)	0.0007 (12)	−0.0165 (13)	−0.0140 (9)
C23	0.0722 (18)	0.0345 (11)	0.0433 (13)	−0.0122 (12)	−0.0156 (12)	−0.0057 (10)
C24	0.0356 (10)	0.0298 (9)	0.0392 (11)	−0.0044 (8)	−0.0063 (9)	−0.0071 (8)
C25	0.0222 (7)	0.0218 (7)	0.0231 (8)	−0.0001 (6)	0.0007 (6)	−0.0040 (6)
C26	0.0249 (8)	0.0321 (9)	0.0237 (8)	0.0010 (7)	0.0035 (7)	0.0013 (7)
C27	0.0357 (10)	0.0358 (10)	0.0285 (9)	−0.0016 (8)	0.0007 (8)	0.0080 (8)
C28	0.0415 (11)	0.0262 (9)	0.0303 (10)	0.0068 (8)	−0.0012 (8)	0.0007 (7)
C29	0.0296 (10)	0.0366 (10)	0.0347 (10)	0.0111 (8)	0.0048 (8)	0.0012 (8)
C30	0.0236 (8)	0.0309 (9)	0.0375 (10)	0.0029 (7)	0.0063 (7)	0.0059 (8)
C31	0.0206 (8)	0.0236 (8)	0.0369 (10)	−0.0040 (6)	−0.0021 (7)	0.0026 (7)
C32	0.0220 (8)	0.0291 (9)	0.0365 (10)	−0.0033 (7)	0.0024 (7)	0.0046 (7)
C33	0.0217 (9)	0.0432 (11)	0.0391 (11)	−0.0056 (8)	0.0002 (8)	0.0109 (9)
C34	0.0312 (10)	0.0446 (12)	0.0523 (14)	−0.0138 (9)	−0.0065 (10)	0.0207 (11)
C35	0.0410 (12)	0.0322 (11)	0.0566 (15)	−0.0151 (9)	−0.0145 (11)	0.0129 (10)
C36	0.0329 (10)	0.0257 (9)	0.0446 (12)	−0.0064 (7)	−0.0091 (9)	0.0038 (8)
C37	0.0288 (9)	0.0221 (7)	0.0282 (9)	0.0017 (7)	−0.0042 (7)	−0.0027 (7)
C38	0.0220 (8)	0.0321 (9)	0.0295 (9)	0.0013 (7)	0.0015 (7)	−0.0059 (7)
C39	0.0203 (8)	0.0304 (9)	0.0304 (9)	−0.0018 (7)	0.0007 (7)	0.0008 (7)
C40	0.0281 (9)	0.0221 (8)	0.0425 (11)	0.0007 (7)	−0.0012 (8)	−0.0031 (8)

### Geometric parameters (Å, °)

**Table:**

Cr1—C37	1.8890 (18)	C11—C12	1.378 (3)
Cr1—C38	1.882 (2)	C11—H11A	0.9500
Cr1—C39	1.888 (2)	C12—H12A	0.9500
Cr1—C40	1.8885 (19)	C13—C14	1.390 (3)
Cr1—P1	2.3333 (5)	C13—C18	1.402 (3)
Cr1—P2	2.3320 (5)	C14—C15	1.383 (3)
P1—C7	1.8330 (18)	C14—H14A	0.9500
P1—C1	1.8382 (17)	C15—C16	1.369 (4)
P1—C13	1.8396 (19)	C15—H15A	0.9500
P2—C31	1.8315 (19)	C16—C17	1.371 (4)
P2—C25	1.8370 (18)	C16—H16A	0.9500
P2—C19	1.8498 (19)	C17—C18	1.381 (3)
F1—C3	1.362 (2)	C17—H17A	0.9500
F1X—C5	1.3991 (10)	C18—H18A	0.9500
F2—C9	1.293 (3)	C19—C20	1.390 (3)
F2X—C11	1.3909 (10)	C19—C24	1.399 (3)
F3—C15	1.320 (3)	C20—C21	1.391 (3)
F3X—C17	1.233 (8)	C20—H20A	0.9500
F4—C21	1.250 (3)	C21—C22	1.372 (4)
F4X—C23	1.3881 (10)	C21—H21A	0.9500
F5—C27	1.320 (3)	C22—C23	1.337 (4)
F5X—C29	1.354 (3)	C22—H22A	0.9500
F6—C33	1.349 (3)	C23—C24	1.356 (3)
F6X—C35	1.3987 (10)	C23—H23A	0.9500
O1—C37	1.144 (2)	C24—H24A	0.9500
O2—C38	1.152 (2)	C25—C26	1.393 (3)
O3—C39	1.145 (2)	C25—C30	1.395 (3)
O4—C40	1.153 (2)	C26—C27	1.378 (3)
C1—C6	1.394 (2)	C26—H26A	0.9500
C1—C2	1.397 (2)	C27—C28	1.380 (3)
C2—C3	1.375 (2)	C27—H27A	0.9500
C2—H2A	0.9500	C28—C29	1.372 (3)
C3—C4	1.376 (3)	C28—H28A	0.9500
C3—H3A	0.9500	C29—C30	1.389 (3)
C4—C5	1.379 (3)	C29—H29A	0.9500
C4—H4A	0.9500	C30—H30A	0.9500
C5—C6	1.393 (3)	C31—C36	1.396 (3)
C5—H5A	0.9500	C31—C32	1.396 (3)
C6—H6A	0.9500	C32—C33	1.380 (3)
C7—C12	1.398 (3)	C32—H32A	0.9500
C7—C8	1.400 (3)	C33—C34	1.370 (3)
C8—C9	1.385 (3)	C33—H33A	0.9500
C8—H8A	0.9500	C34—C35	1.376 (4)
C9—C10	1.373 (3)	C34—H34A	0.9500
C9—H9A	0.9500	C35—C36	1.391 (3)
C10—C11	1.351 (3)	C35—H35A	0.9500
C10—H10A	0.9500	C36—H36A	0.9500
			
C38—Cr1—C39	170.47 (9)	C15—C16—C17	117.6 (2)
C38—Cr1—C40	95.03 (9)	C15—C16—H16A	121.2
C39—Cr1—C40	94.50 (9)	C17—C16—H16A	121.2
C38—Cr1—C37	85.09 (8)	F3X—C17—C16	104.6 (4)
C39—Cr1—C37	85.39 (8)	F3X—C17—C18	133.7 (5)
C40—Cr1—C37	179.59 (9)	C16—C17—C18	121.3 (2)
C38—Cr1—P2	89.85 (6)	C16—C17—H17A	119.3
C39—Cr1—P2	90.23 (6)	C18—C17—H17A	119.3
C40—Cr1—P2	87.42 (6)	C17—C18—C13	120.3 (2)
C37—Cr1—P2	92.97 (6)	C17—C18—H18A	119.8
C38—Cr1—P1	90.28 (6)	C13—C18—H18A	119.8
C39—Cr1—P1	90.51 (6)	C20—C19—C24	119.08 (18)
C40—Cr1—P1	87.35 (6)	C20—C19—P2	122.15 (15)
C37—Cr1—P1	92.26 (6)	C24—C19—P2	118.76 (15)
P2—Cr1—P1	174.760 (19)	C19—C20—C21	119.1 (2)
C7—P1—C1	105.07 (8)	C19—C20—H20A	120.4
C7—P1—C13	101.15 (9)	C21—C20—H20A	120.4
C1—P1—C13	98.60 (8)	F4—C21—C22	114.0 (2)
C7—P1—Cr1	114.52 (6)	F4—C21—C20	123.3 (3)
C1—P1—Cr1	115.96 (6)	C22—C21—C20	122.5 (2)
C13—P1—Cr1	119.13 (6)	C22—C21—H21A	118.8
C31—P2—C25	104.54 (8)	C20—C21—H21A	118.8
C31—P2—C19	101.37 (9)	C23—C22—C21	115.1 (2)
C25—P2—C19	99.18 (8)	C23—C22—H22A	122.4
C31—P2—Cr1	113.17 (6)	C21—C22—H22A	122.4
C25—P2—Cr1	116.74 (6)	C22—C23—C24	127.3 (2)
C19—P2—Cr1	119.49 (7)	C22—C23—F4X	109.5 (2)
C6—C1—C2	118.91 (16)	C24—C23—F4X	123.1 (3)
C6—C1—P1	126.21 (14)	C22—C23—H23A	116.4
C2—C1—P1	114.86 (13)	C24—C23—H23A	116.4
C3—C2—C1	118.76 (17)	C23—C24—C19	116.9 (2)
C3—C2—H2A	120.6	C23—C24—H24A	121.6
C1—C2—H2A	120.6	C19—C24—H24A	121.6
F1—C3—C2	117.33 (17)	C26—C25—C30	118.77 (17)
F1—C3—C4	119.14 (17)	C26—C25—P2	116.23 (13)
C2—C3—C4	123.53 (18)	C30—C25—P2	124.99 (14)
C2—C3—H3A	118.2	C27—C26—C25	119.77 (18)
C4—C3—H3A	118.2	C27—C26—H26A	120.1
C3—C4—C5	117.32 (17)	C25—C26—H26A	120.1
C3—C4—H4A	121.3	F5—C27—C26	115.3 (2)
C5—C4—H4A	121.3	F5—C27—C28	121.9 (2)
C4—C5—C6	121.21 (18)	C26—C27—C28	122.39 (19)
C4—C5—F1X	126.6 (9)	C26—C27—H27A	118.8
C6—C5—F1X	109.3 (9)	C28—C27—H27A	118.8
C4—C5—H5A	119.4	C29—C28—C27	117.25 (18)
C6—C5—H5A	119.4	C29—C28—H28A	121.4
C5—C6—C1	120.21 (18)	C27—C28—H28A	121.4
C5—C6—H6A	119.9	F5X—C29—C28	119.4 (2)
C1—C6—H6A	119.9	F5X—C29—C30	117.7 (2)
C12—C7—C8	118.88 (17)	C28—C29—C30	122.41 (19)
C12—C7—P1	120.90 (15)	C28—C29—H29A	118.8
C8—C7—P1	120.00 (14)	C30—C29—H29A	118.8
C9—C8—C7	118.66 (19)	C29—C30—C25	119.36 (18)
C9—C8—H8A	120.7	C29—C30—H30A	120.3
C7—C8—H8A	120.7	C25—C30—H30A	120.3
F2—C9—C10	116.36 (19)	C36—C31—C32	119.11 (18)
F2—C9—C8	120.4 (2)	C36—C31—P2	120.82 (16)
C10—C9—C8	123.3 (2)	C32—C31—P2	119.76 (14)
C10—C9—H9A	118.4	C33—C32—C31	118.43 (19)
C8—C9—H9A	118.4	C33—C32—H32A	120.8
C11—C10—C9	116.27 (19)	C31—C32—H32A	120.8
C11—C10—H10A	121.9	F6—C33—C34	118.09 (19)
C9—C10—H10A	121.9	F6—C33—C32	118.4 (2)
C10—C11—C12	124.5 (2)	C34—C33—C32	123.5 (2)
C10—C11—F2X	112.3 (3)	C34—C33—H33A	118.3
C12—C11—F2X	123.1 (3)	C32—C33—H33A	118.3
C10—C11—H11A	117.8	C33—C34—C35	117.8 (2)
C12—C11—H11A	117.8	C33—C34—H34A	121.1
C11—C12—C7	118.4 (2)	C35—C34—H34A	121.1
C11—C12—H12A	120.8	C34—C35—C36	121.0 (2)
C7—C12—H12A	120.8	C34—C35—F6X	106.2 (12)
C14—C13—C18	118.79 (19)	C36—C35—F6X	132.4 (12)
C14—C13—P1	121.97 (16)	C34—C35—H35A	119.5
C18—C13—P1	119.22 (15)	C36—C35—H35A	119.5
C15—C14—C13	118.4 (2)	C35—C36—C31	120.1 (2)
C15—C14—H14A	120.8	C35—C36—H36A	119.9
C13—C14—H14A	120.8	C31—C36—H36A	119.9
F3—C15—C16	118.0 (2)	O1—C37—Cr1	179.42 (18)
F3—C15—C14	118.5 (3)	O2—C38—Cr1	174.59 (18)
C16—C15—C14	123.5 (2)	O3—C39—Cr1	176.02 (18)
C16—C15—H15A	118.2	O4—C40—Cr1	179.6 (2)
C14—C15—H15A	118.2		
			
C38—Cr1—P1—C7	−33.20 (9)	C18—C13—C14—C15	1.3 (3)
C39—Cr1—P1—C7	156.30 (9)	P1—C13—C14—C15	−177.03 (17)
C40—Cr1—P1—C7	61.82 (10)	C13—C14—C15—F3	−179.9 (2)
C37—Cr1—P1—C7	−118.30 (9)	C13—C14—C15—C16	−0.8 (4)
C38—Cr1—P1—C1	89.47 (9)	F3—C15—C16—C17	178.7 (2)
C39—Cr1—P1—C1	−81.03 (9)	C14—C15—C16—C17	−0.3 (4)
C40—Cr1—P1—C1	−175.51 (9)	C15—C16—C17—F3X	175.5 (5)
C37—Cr1—P1—C1	4.38 (9)	C15—C16—C17—C18	1.0 (4)
C38—Cr1—P1—C13	−153.04 (9)	F3X—C17—C18—C13	−173.1 (7)
C39—Cr1—P1—C13	36.46 (9)	C16—C17—C18—C13	−0.5 (3)
C40—Cr1—P1—C13	−58.02 (9)	C14—C13—C18—C17	−0.7 (3)
C37—Cr1—P1—C13	121.86 (9)	P1—C13—C18—C17	177.71 (17)
C38—Cr1—P2—C31	157.99 (9)	C31—P2—C19—C20	−114.30 (18)
C39—Cr1—P2—C31	−31.54 (9)	C25—P2—C19—C20	138.73 (18)
C40—Cr1—P2—C31	62.95 (10)	Cr1—P2—C19—C20	10.8 (2)
C37—Cr1—P2—C31	−116.93 (9)	C31—P2—C19—C24	66.35 (18)
C38—Cr1—P2—C25	−80.59 (9)	C25—P2—C19—C24	−40.62 (18)
C39—Cr1—P2—C25	89.88 (9)	Cr1—P2—C19—C24	−168.57 (14)
C40—Cr1—P2—C25	−175.63 (9)	C24—C19—C20—C21	0.3 (3)
C37—Cr1—P2—C25	4.49 (9)	P2—C19—C20—C21	−179.09 (19)
C38—Cr1—P2—C19	38.77 (9)	C19—C20—C21—F4	−173.8 (3)
C39—Cr1—P2—C19	−150.76 (9)	C19—C20—C21—C22	0.3 (4)
C40—Cr1—P2—C19	−56.27 (10)	F4—C21—C22—C23	173.7 (3)
C37—Cr1—P2—C19	123.84 (9)	C20—C21—C22—C23	−0.9 (4)
C7—P1—C1—C6	2.00 (19)	C21—C22—C23—C24	1.0 (4)
C13—P1—C1—C6	106.08 (17)	C21—C22—C23—F4X	177.3 (3)
Cr1—P1—C1—C6	−125.52 (16)	C22—C23—C24—C19	−0.5 (4)
C7—P1—C1—C2	−176.57 (13)	F4X—C23—C24—C19	−176.3 (3)
C13—P1—C1—C2	−72.49 (14)	C20—C19—C24—C23	−0.2 (3)
Cr1—P1—C1—C2	55.91 (14)	P2—C19—C24—C23	179.17 (17)
C6—C1—C2—C3	−0.2 (3)	C31—P2—C25—C26	177.34 (14)
P1—C1—C2—C3	178.51 (14)	C19—P2—C25—C26	−78.29 (15)
C1—C2—C3—F1	−177.86 (16)	Cr1—P2—C25—C26	51.49 (15)
C1—C2—C3—C4	2.2 (3)	C31—P2—C25—C30	−4.31 (19)
F1—C3—C4—C5	177.74 (18)	C19—P2—C25—C30	100.06 (17)
C2—C3—C4—C5	−2.3 (3)	Cr1—P2—C25—C30	−130.16 (15)
C3—C4—C5—C6	0.4 (3)	C30—C25—C26—C27	1.4 (3)
C3—C4—C5—F1X	−158.2 (11)	P2—C25—C26—C27	179.82 (15)
C4—C5—C6—C1	1.4 (3)	C25—C26—C27—F5	−172.4 (2)
F1X—C5—C6—C1	163.4 (9)	C25—C26—C27—C28	0.8 (3)
C2—C1—C6—C5	−1.6 (3)	F5—C27—C28—C29	170.7 (2)
P1—C1—C6—C5	179.92 (15)	C26—C27—C28—C29	−2.1 (3)
C1—P1—C7—C12	128.51 (15)	C27—C28—C29—F5X	−170.5 (2)
C13—P1—C7—C12	26.33 (17)	C27—C28—C29—C30	1.1 (3)
Cr1—P1—C7—C12	−103.10 (15)	F5X—C29—C30—C25	172.8 (2)
C1—P1—C7—C8	−56.90 (16)	C28—C29—C30—C25	1.0 (3)
C13—P1—C7—C8	−159.07 (15)	C26—C25—C30—C29	−2.2 (3)
Cr1—P1—C7—C8	71.50 (15)	P2—C25—C30—C29	179.47 (16)
C12—C7—C8—C9	−1.5 (3)	C25—P2—C31—C36	132.33 (16)
P1—C7—C8—C9	−176.25 (14)	C19—P2—C31—C36	29.60 (18)
C7—C8—C9—F2	−176.82 (19)	Cr1—P2—C31—C36	−99.61 (16)
C7—C8—C9—C10	1.6 (3)	C25—P2—C31—C32	−54.22 (17)
F2—C9—C10—C11	177.87 (19)	C19—P2—C31—C32	−156.94 (16)
C8—C9—C10—C11	−0.6 (3)	Cr1—P2—C31—C32	73.85 (16)
C9—C10—C11—C12	−0.4 (3)	C36—C31—C32—C33	−1.5 (3)
C9—C10—C11—F2X	−176.2 (3)	P2—C31—C32—C33	−175.10 (15)
C10—C11—C12—C7	0.4 (3)	C31—C32—C33—F6	−178.06 (17)
F2X—C11—C12—C7	175.7 (3)	C31—C32—C33—C34	1.7 (3)
C8—C7—C12—C11	0.6 (3)	F6—C33—C34—C35	179.32 (19)
P1—C7—C12—C11	175.25 (15)	C32—C33—C34—C35	−0.5 (3)
C7—P1—C13—C14	−116.34 (17)	C33—C34—C35—C36	−1.0 (3)
C1—P1—C13—C14	136.33 (17)	C33—C34—C35—F6X	−175.0 (12)
Cr1—P1—C13—C14	10.10 (19)	C34—C35—C36—C31	1.1 (3)
C7—P1—C13—C18	65.34 (16)	F6X—C35—C36—C31	173.3 (16)
C1—P1—C13—C18	−41.99 (16)	C32—C31—C36—C35	0.2 (3)
Cr1—P1—C13—C18	−168.22 (13)	P2—C31—C36—C35	173.69 (16)

### Hydrogen-bond geometry (Å, °)

**Table:**

*D*—H···*A*	*D*—H	H···*A*	*D*···*A*	*D*—H···*A*
C2—H2A···O2^i^	0.95	2.52	3.105 (2)	120
C4—H4A···F1^ii^	0.95	2.41	3.292 (2)	153
C10—H10A···F4^iii^	0.95	2.53	3.271 (3)	135
C28—H28A···F5^iv^	0.95	2.33	3.069 (3)	135
C34—H34A···F3^v^	0.95	2.32	3.191 (3)	153

Symmetry codes: (i) *x*, −*y*+1/2, *z*+1/2; (ii) −*x*+1, −*y*+1, −*z*+1; (iii) −*x*+1, −*y*, −*z*; (iv) −*x*, −*y*+1, −*z*; (v) −*x*, −*y*, −*z*+1.
